# Efficacy of Polyunsaturated Fatty Acids on Inflammatory Markers in Patients Undergoing Dialysis: A Systematic Review with Network Meta-Analysis of Randomized Clinical Trials

**DOI:** 10.3390/ijms20153645

**Published:** 2019-07-25

**Authors:** Po-Kuan Wu, Shu-Ching Yeh, Shan-Jen Li, Yi-No Kang

**Affiliations:** 1 School of Medicine, College of Medicine, Taipei Medical University, Taipei 11042, Taiwan; 2 Division of Nephrology, Department of Internal Medicine, Taipei Medical University Hospital, Taipei 11042, Taiwan; 3 Department of Emergency Medicine, Taipei Medical University Hospital, Taipei 11042, Taiwan; 4 Evidence-Based Medicine Center, Wan Fang Hospital, Taipei Medical University, Taipei 11696, Taiwan

**Keywords:** polyunsaturated fatty acids, omega-3 fatty acid, inflammatory maker, C-reactive protein, interleukin-6

## Abstract

The effects of polyunsaturated fatty acids (PUFAs) on inflammatory markers among patients receiving dialysis have been discussed for a long time, but previous syntheses made controversial conclusion because of highly conceptual heterogeneity in their synthesis. Thus, to further understanding of this topic, we comprehensively gathered relevant randomized clinical trials (RCTs) before April 2019, and two authors independently extracted data of C-reactive protein (CRP), high-sensitivity C-reactive protein (hs-CRP), and interleukin-6 (IL-6) for conducting network meta-analysis. Eighteen eligible RCTs with 962 patients undergoing dialysis were included in our study. The result showed that with placebo as the reference, PUFAs was the only treatment showing significantly lower CRP (weighted mean difference (WMD): −0.37, 95% confidence interval (CI): −0.07 to −0.68), but the CRP in PUFAs group was not significantly lower than vitamin E, PUFAs plus vitamin E, or medium-chain triglyceride. Although no significant changes were noted for hs-CRP and IL-6 levels, PUFAs showed the best ranking among treatments according to surface under the cumulative ranking. Therefore, PUFAs could be a protective option for patients receiving dialysis in clinical practice.

## 1. Introduction

Patients with end-stage renal disease around the world suffer from chronic inflammation caused by dialysis, especially hemodialysis. For instance, vascular access of hemodialysis [1], filter membrane of hemodialysis machine [2], and endotoxin from the dialysate [3] all cause chronic inflammation. Chronic inflammation not only reduces the quality-of-life among patients with chronic kidney disease, but also increases the mortality rate of these populations [4,5]. Previous reports have noted about 30% to 50% of patients undergoing hemodialysis have activated inflammatory response [6]. Chronic inflammation is related to pathogenesis of atherosclerosis [7], and cardiovascular disease accounts for the largest proportion of mortality in patients with chronic kidney disease [8]. As a result, detecting Interleukin-6 (IL-6), C-reactive protein (CRP), and high-sensitivity C-reactive protein (hs-CRP) are imperative for understanding and managing inflammatory conditions among these patients [4,5]. The association between IL-6, CRP, and cardiovascular disease is well known. Also, hs-CRP is another important marker manifesting inflammation and endothelial damage; it is also an indicator of defective coronary artery blood flow [9].

To improve the outcomes among patients with hemodialysis, controlling their inflammatory status is an important aspect in clinical practice. In the past decade, many trials have tried to control these inflammations among this group of patients through nutrient supplements [10,11,12,13,14,15,16,17,18,19,20,21,22,23,24,25,26,27,28,29,30]. Commonly-used nutrient supplements such as polyunsaturated fatty acids (PUFAs), vitamin D, antioxidants, polyphenol-rich foods, fibers, and probiotics to modulate patient’s immune response are becoming more and more popular [31]. Unlike consuming drugs, nutrient supplements are usually free from increment of patients’ physical stress. PUFAs are the top two popular nutrient supplements with many trials and Omega-3 fatty acids are the main nutrients as trials used PUFAs for reducing inflammatory among patients with chronic kidney disease [31]. The association of PUFA and inflammatory processes has been widely discussed in the past decade [32,33,34,35,36,37,38,39,40]. With regard to Omega-3 fatty acids, it commonly involves eicosapentaenoic acid, docosahexaenoic acid, and alpha-linolenic acid. Eicosapentaenoic acid and docosahexaenoic acid upregulate peroxisome proliferators’ active receptors, and decrease CRP and IL-6. The peroxisome proliferators’ active receptors also decrease CRP and IL-6 through downregulation of nuclear factor kappa B (NF-κB) [41,42].

A good synthesis for the topic of nutrients on inflammatory markers among patients with hemodialysis in 2018, yet there is a very high heterogeneity (I-square = 84.3%) existing in the result of CRP mean changes after Omega-3 fatty acids supplementation [31]. Therefore, this topic needs further analysis to provide a clearer picture for the effects of PUFAs on inflammatory markers among patients undergoing hemodialysis. Moreover, there is another meta-analysis in 2018 showing that alpha-linolenic acid cannot affect relevant inflammatory markers [43]. Therefore, our study aimed to clarify whether using PUFAs can reduce inflammatory cytokines (CRP, IL-6, hs-CRP) among patients undergoing dialysis through systematic review and meta-analysis of randomized clinical trials.

## 2. Results

**Figure 1:**
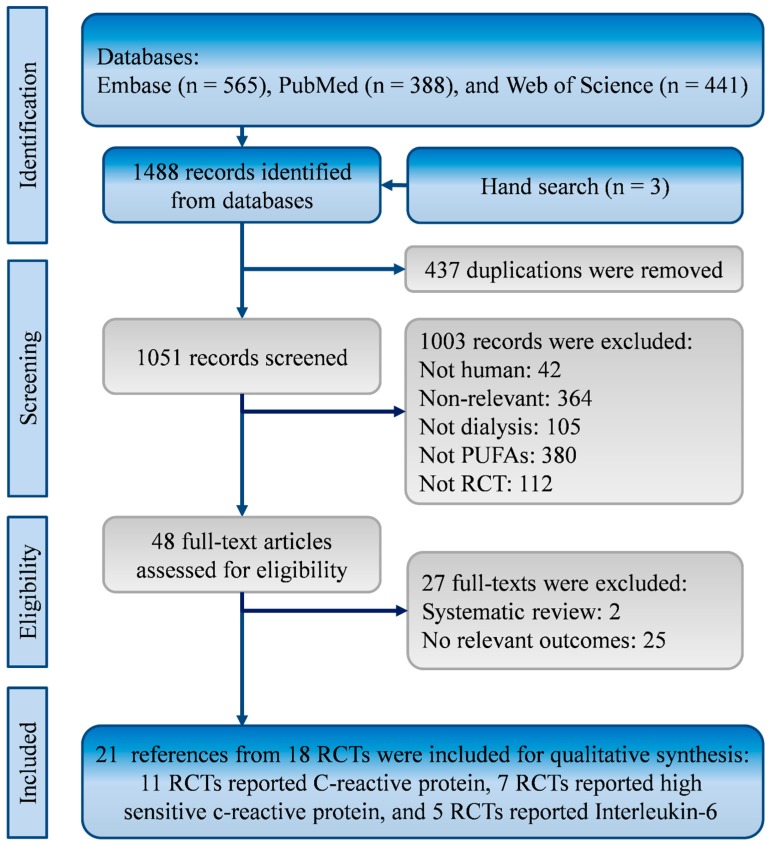
Flowchart of the systematic review and meta-analysis according to PRISMA guidelines.

Through comprehensive search, this systematic review identified 1485 references from the EMBASE (*n* = 656), PubMed (*n* = 388), and Web of Science (*n* = 441). Three records were found from hand search of reference lists. After the exclusion of 437 duplications, 1051 references were reviewed for eligibility. Then, two systematic reviews and 25 randomized clinical trials without relevant outcomes were excluded [31,44,45,46,47,48,49,50,51,52,53,54,55,56,57,58,59,60,61,62,63,64,65,66,67,68,69]. There were 21 references from 18 RCTs meeting the eligibility criteria (Figure 1) [10,11,12,13,14,15,16,17,18,19,20,21,22,23,24,25,26,27,28,29,30].

### 2.1. Characteristics and Quality of Included Studies

The 18 trials recruited 962 patient undergoing dialysis from Brazil [24], Denmark [13,18], Greece [27], Egypt [30], Iran [10,14,21,22,25,26], Korea [23], United States [11,12,19,20,29], and Sweden [28]. The treatments in the 18 trials could be categorized into five treating strategies including placebo, PUFAs, vitamin E, medium chain triglyceride (MCT), and PUFAs plus vitamin E. The available data in each study showed mean ages from 46.4 to 68 years old, and a total of 598 (62.2%) men were included in the studies. Other information about trial location, inclusion years, treatments, number of patients, and dialysis period are shown in Table 1. Overall, the quality of the studies is presented in Table 2. 

### 2.2. C-Reactive Protein

**Figure 2:**
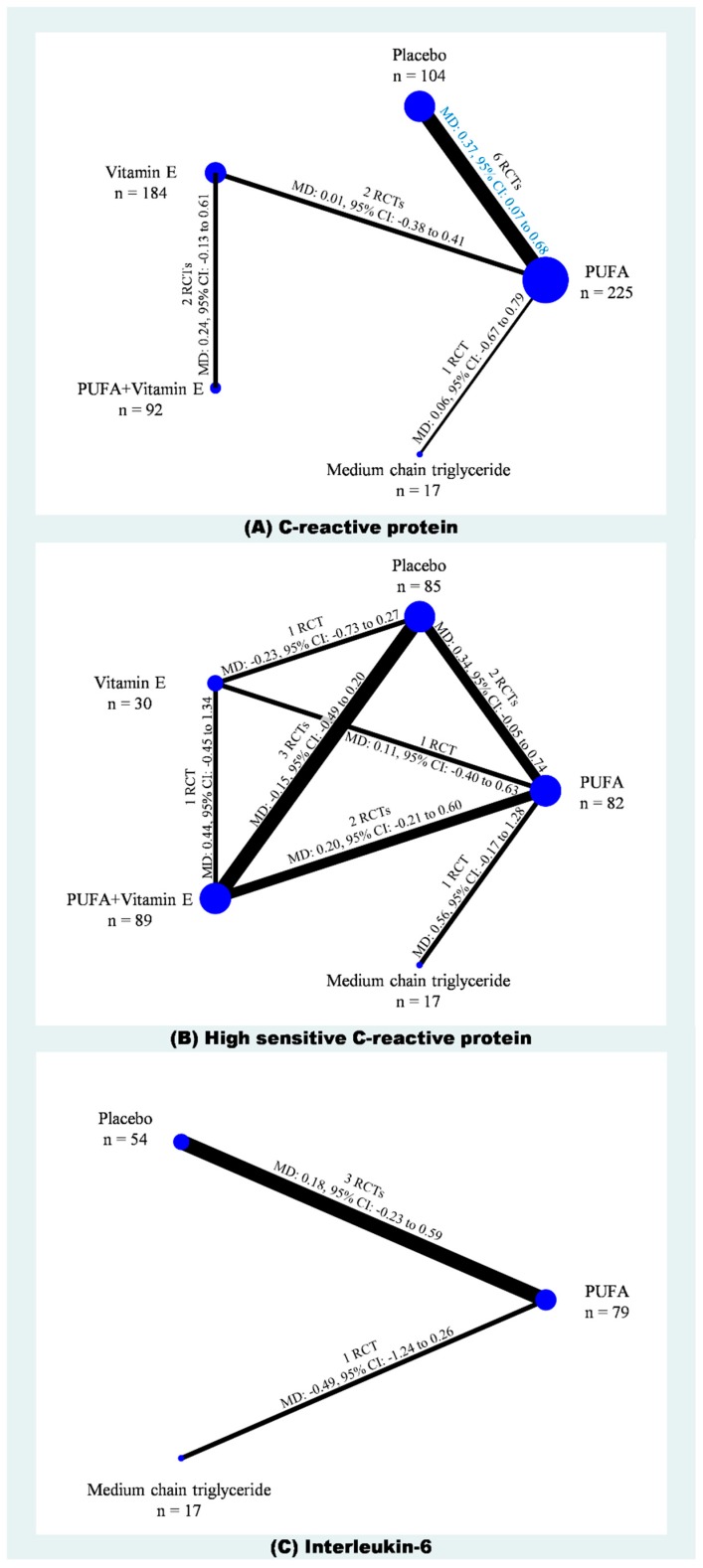
Network plots of (**A**) CRP, (**B**) high sensitivity CRP, and (**C**) IL-6.

**Figure 3:**
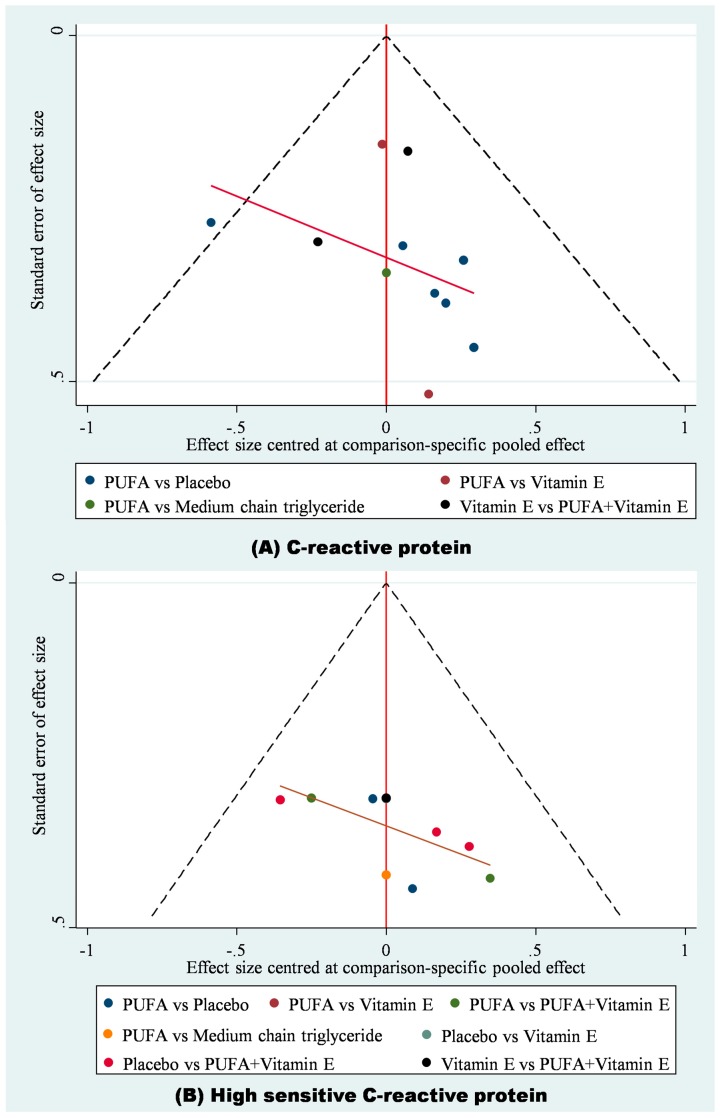
Funnel plots of (**A**) CRP and (**B**) high sensitivity CRP.

A total of 11 RCTs with 632 cases in five treatments were included in the network meta-analysis of CRP (Figure 2A) [12,13,14,18,21,22,23,24,26,27,29]. The result showed that with placebo as the reference, PUFAs was the only one treatment showing significantly lower CRP (WMD: −0.37, 95% CI: −0.07 to −0.68), but the CRP in PUFAs group was not significantly lower than vitamin E, PUFAs plus vitamin E, and MCT (Figure 3A; Supplementary File 1). Similarly, in SUCRA, PUFAs also had the highest value (Mean rank = 2.1; SUCRA = 72.7) and placebo had the lowest value (Mean rank = 4.3; SUCRA = 16.3; Supplementary File 2). Because placebo, PUFA, vitamin E, PUFAs plus vitamin E, and MCT did not form any loop in the network meta-analysis of CRP, it is not required to test inconsistency in this consistency model. Moreover, no evidence detected serious small study effects (*t* = 0.49, 95% CI: −1.27 to 1.98; Supplementary File 3).

### 2.3. High-Sensitivity C-Reactive Protein

Six trials provided data on hs-CRP among the five treatments (*n* = 303) (Figure 2B) [10,11,20,25,28,30]. According to the available data, our network meta-analysis of hs-CRP showed no significant differences among placebo, PUFA, vitamin E, PUFAs plus vitamin E, and MCT (Supplementary File 4). There were some hints from ranking the best hs-CRP probability showing that PUFAs (54.7%) was the best choice, whereas placebo (1.2%) should be avoided. Similarly, in SUCRA, PUFAs had the highest value (Mean rank = 1.6; SUCRA = 84.6), whereas placebo (Mean rank = 3.9; SUCRA = 28.2) and MCT (Mean rank = 4.3; SUCRA = 18.4; Supplementary File 5) were the two treatments with lowest values. The loop inconsistency test for the network meta-analysis of hs-CRP showed insignificance (chi-square = 2.25, *p* < 0.13; Supplementary File 6), yet the Egger’s test detected small study effects (*t* = 2.34, 95% CI: 0.10 to 6.06, *p* = 0.04; Supplementary File 7).

### 2.4. Interleukin-6

Four of the included trials provided data on IL-6 among placebo, PUFA, and MCT (*n* = 140) (Figure 2A) [14,20,22,26]. The network meta-analysis of IL-6 also showed no significant differences among placebo, PUFA, and MCT (Supplementary File 8). Interestingly, ranking the best IL-6 probability showing that MCT (88.8%) had the highest probability, whereas placebo (3.2%) and PUFAs (8.0%) were the two treatments with lowest probabilities. SUCRA also depicted similar phenomenon showing that MCT had the highest value (Mean rank = 1.2; SUCRA = 92.0), whereas placebo (Mean rank = 2.7; SUCRA = 12.7) and MCT (Mean rank = 2.1; SUCRA = 45.2) were the two treatments with lowest values. The loop inconsistency test for the network meta-analysis of hs-CRP showed no significance (chi-square = 1.64, *p* < 0.20; Supplementary File 9), and the Egger’s test also detected no small study effects (*t* = 1.44, 95% CI: −14.26 to 28.63, *p* = 0.29; Supplementary File 10).

### 2.5. Further Analysis

We further examined albumin, a minor parameter of inflammatory, based on the included evidence, and we also detected the influences from regions because of lifestyle and dietary style. For albumin, A total of nine trials formed network for placebo, PUFA, vitamin E, and PUFAs plus vitamin E with 407 cases [10,12,13,15,20,23,27,28,29]. The pooled result also showed insignificant differences among those four treatments (Supplementary File 11). Yet, SUCRA indicated that PUFA had the highest value (Mean rank = 1.4, SUCRA = 86.9; Supplementary File 12). We did not observe significant inconsistency (chi-square = 5.38, *p* < 0.06; Supplementary File 13) and small study effects (*t* = 1.02, 95% CI: −1.69 to 4.65, *p* = 0.33; Supplementary File 14).

Moreover, we only found available and appropriate information for detecting region effects on the pooled results of CRP and hs-CRP. Then, the meta-regression did not show any significant findings. Current evidence is insufficient to prove that region plays an important role in the effects of PUFA on CRP (Supplementary File 15) and hs-CRP (Supplementary File 16).

A cluster plot for CRP and hs-CRP scores demonstrated that the best balance was achieved by PUFAs (Cophenetic Correlation Coefficient = 0.86; Supplementary File 17). Therefore, PUFAs may be recommended for treating patients undergoing dialysis. By contrast, placebo exhibited poor performance in the cluster plot. 

## 3. Discussion

In our systematic review, we successfully identified 18 randomized clinical trials (*n* = 962) investigating the effect of PUFAs on inflammatory markers among patients undergoing dialysis. In our network meta-analysis, we depicted an overview of comparisons among placebo, PUFA, vitamin E, PUFAs plus vitamin E, and MCT. Then, the results showed that the current evidence only supports PUFAs group having significant lower CRP than placebo. Yet, other active treatments did not reach the statistical significance when they were compared to placebo. The results of hs-CRP and IL-6 failed to support PUFAs having more benefits than other treatment, even no significant benefit as it was compared to placebo. These results about limited anti-inflammatory effects from PUFAs are similar to a previous synthesis indicating that alpha-linolenic acid has no effects on blood inflammatory markers [43].

These insignificant results may relate to the complex comparators among trials. Some of the trials treated control group with vitamin E, and some of them used combined supplements of PUFAs and vitamin E. Apart from PUFA, antioxidants such as vitamin E can also reduce the inflammatory responses by decreasing reactive oxygen species and NF-κB [70]. Besides, combination of PUFAs and antioxidants can reduce the oxidative stress caused by PUFAs [1]. Thus, our synthesis separated PUFAs alone and combined supplements of PUFAs and vitamin E. Then, we found PUFAs alone having a significantly higher CRP level than placebo, whereas no significant difference in CRP level between combined supplements of PUFAs and vitamin E and placebo. The pooled result also did not support that combined supplements of PUFAs and vitamin E having significant benefits on hs-CRP.

We agree that Omega-3 fatty acids play some roles in anti-inflammatory among patients undergoing dialysis because eicosapentaenoic acid and docosahexaenoic acid have been well-known in anti-inflammatory action through regulating gene expression, lowering membrane content of arachidonic acid, inhibiting arachidonic acid metabolism, and competing with arachidonic acid [41,71]. To be more specific, eicosapentaenoic acid and docosahexaenoic acid affect cyclooxygenase and lipoxygenase pathway through being substrates for the key enzymes. The patients of chronic kidney disease have much higher systemic concentration of inflammatory cytokine because of the decrease renal clearance and the insufficiency of nutrient [72,73]. This situation will cause the destruction of endothelial cell and eventually lead to cardiovascular disease [72,74]. To control the chronic inflammation of these patients, dietary fat supplement has important biological effect. PUFA can clean up the reactive oxygen species and inhibit activation of NF-κB, which plays a significant role in regulating inflammatory response [31]. Furthermore, PUFA will compete with arachidonic acid for the substance in the cyclooxygenase pathway to produce less pro-inflammatory cytokine [41]. Collectively, PUFA can decrease the systemic inflammation and the cardiovascular disease mortality among patients with chronic kidney disease. Although our evidence did not show the significant benefit of Omega-3 fatty acids on hs-CRP among patients receiving dialysis, the pooled hs-CRP had similar trends with the pooled results of CRP. A potential reason for the insignificant difference in hs-CRP may be smaller sample size (n = 303). In addition, another important potential factor causing the insignificant finding is that hs-CRP detects inflammatory with better sensitivity than CRP, especially for those patients with cardiovascular problems. It is well-published that cardiovascular problems are common comorbidities of chronic kidney disease. Thus, the difference between hs-CRP and traditional CRP may be more obvious for patients undergoing dialysis than for healthy people. 

### 3.1. Comparing to the Previous Syntheses

Besides the good systematic review and meta-analysis in 2018 we mentioned above [31], there is another important synthesis in 2016 on this topic [69]. These two systematic reviews concluded similarly by declaring that Omega-3 fatty acids are effective supplements for reducing CRP levels among patients undergoing dialysis. However, their meta-analyses of CRP reflected very high heterogeneities. They did not successfully explain the source of heterogeneities though the systematic review by Khor et al. in 2018 separated alpha-linolenic acid (I-square = 93.4%) from eicosapentaenoic acid and docosahexaenoic acid. As we know, the anti-inflammatory effects of alpha-linolenic acid share similar pathway with eicosapentaenoic acid and docosahexaenoic acid [31]. Thus, subgroup analysis for alpha-linolenic acid may be not the best way to explore the heterogeneity in the pooled result of CRP mean change. 

To face the challenge of high heterogeneity in the pooled CRP reported by the previous syntheses, in our study, we carefully clarified comparators by mainly relevant nutrients and outcomes for giving fewer biased results because of conceptual heterogeneity according to methodological guidance [75]. These two conceptual heterogeneities may result in the statistical heterogeneity in the pooled CRP level. Our study not only distinguished placebo, PUFA, vitamin E, PUFAs plus vitamin E, and MCT for comparators, but also separated hs-CRP from CRP for outcome synthesis. For instance, four trials included in the previous synthesis used hs-CRP [10,11,20,25], and we pooled these four trials with the other two trials that were not included in the previous synthesis for hs-CRP [28,30]. As a result of reduction of conceptual heterogeneity, we successfully gave this topic reasonable results without inconsistency and highly statistical heterogeneity. 

### 3.2. Limitations

Although our synthesis overcame some limitations in previous syntheses and clarified the effects of PUFAs on inflammatory markers among patients receiving dialysis, the present synthesis still has three limitations. Firstly, the use of PUFAs is not clear, though our study separated combined supplements of PUFAs and vitamin E from PUFAs alone. The separation resulted in lower heterogeneity than previous syntheses, but our study cannot make a practical suggestion with a specific dosage for the use of PUFAs. Secondly, interaction of PUFAs and vitamin E on the anti-inflammatory effects remains unclear. Our synthesis did not find better results as the trials treating patients with combined supplements of PUFAs and vitamin E. Thirdly, our synthesis showed some trends about the benefits of PUFAs on hs-CRP and IL-6, but the results may be under power because of small sample size. We suggest that future studies should use hs-CRP measurement to confirm whether using PUFAs can reduce inflammatory responses, especially among those patients undergoing dialysis. 

## 4. Materials and Methods

This comprehensive review team consisting of nephrologists and an experienced researcher conducted this study according to the Cochrane handbook, and reported the systematic review and meta-analysis according to the PRISMA guidelines [76]. The experienced researcher previously participated in some studies about nutrient, internal medicine, and chronic kidney disease [77,78,79,80]. The researcher also has some experience in conducting network meta-analysis [81,82]. Because this meta-analysis uses published data, it was exempted from institutional review board approval.

### 4.1. Study Selection Criteria

According to our study purpose, this comprehensive review selected evidence if (1) the study recruited patients undergoing dialysis, (2) the intervention was PUFA, and (3) the study prospectively randomized patients into two or more groups. However, this comprehensive review removed studies when they met following exclusion criteria: (1) the reference was gray literature without detailed information or data, (2) the study did not separate outcome reporting as it concurrently recruited patients with and without dialysis, and (3) the article did not report any relevant outcomes (CRP, hs-CRP, and IL-6). 

### 4.2. Search Strategy and Study Selection

Data sources were three important online databases including EMBASE, PubMed, and the Web of Science. PubMed was the platform for building search strategy with relevant terms of dialysis and PUFA, and the search strategy was adapted to the other two databases. The relevant terms involved free-text and medical subject heading. Boolean operator “OR” combined the relevant terms of dialysis, and we also used “OR” for combining relevant terms of PUFA. Then, Boolean operator “AND” connected both dialysis part and PUFAs part. This search strategy did not restrict language and publication date from database inception until April 2019. Supplemental Material 18 showed the detail of the searching strategy.

After relevant references were identified from online databases, two investigators excluded ineligible references according to criteria in two phases. The first phase was title and abstract screening, and the second phase was full-text review. Any references meeting exclusion criteria were removed.

### 4.3. Quality Assessment and Data Extraction

The two investigators independently identified relevant information, and extracted outcome data. They identified the data about the details of trial design, location, inclusion year, treatments, sample size, mean age, sex, and dialysis period. The outcome data included three inflammatory markers, namely CRP, hs-CRP, IL-6, and albumin at the end of treatment. Because these data were continuous, the investigators extracted them in mean and standard deviation (SD). This network meta-analysis estimated SD from standard error (SE) according to the formula SE = SD/√N when the trial only provided SE. When the trial only presented interquartile range (IQR), this study estimated SD using formula IQR/1.35. Moreover, the network meta-analysis estimated SD from maximum and minimum according to Hozo’s method [83]. 

Based on the identified information, the investigators completed the risk of bias in each trial. The assessment involved randomization, concealment, blinding, follow-up duration, loss follow-up, and analysis type. These items reflected selection bias, performance bias, detection bias, and attrition bias. In case of any disagreements on risk of bias between the two investigators, a third reviewer participated into discussion to resolve the disagreement.

### 4.4. Evidence Synthesis and Statistical Analysis

Evidence synthesis consisted of qualitative and quantitative parts. The quantitative synthesis was contrast-based network meta-analysis. Because of conceptual heterogeneity among trial design, the network meta-analysis should be in random-effects model. The main outcomes were CRP, hs-CRP, and IL-6 at the end of treatment. Thus, the analysis performed weighted mean difference (WMD) and 95% confidence interval (CI). Standardized mean difference was the solution for units of measurement including mg/L, mg/dL, ng/L, ng/mL, and pg/mL. To clarify the effects among active treatments, the quantitative synthesis also showed surface under the cumulative ranking (SUCRA). This statistical technique estimated the probability of each treatment among the most effective treatments, and formed a hierarchy through the treatment ranking of probability. We would like to foster the understanding on this topic, and therefore we further analyzed albumin and the influence from region. The influence from region was detected by using meta-regression in network meta-analysis model. For conducting meta-regression, we applied dummy variables for America, Asia, and Europe. To confirm the quality of the quantitative synthesis, the network meta-analysis detected both the small-study effect and inconsistency. The small-study effect in a network meta-analysis can be assessed by an adjusted funnel plot and Egger’s regression intercept. Concerning inconsistency, the meta-analysis implemented Lu–Ades’ loop inconsistency test. The analyses mentioned above were completed using STATA version 14 for Microsoft Windows. In all analysis, *p* < 0.05 was considered as statistically significant. 

## 5. Conclusions

Based on available evidence, the very first network meta-analysis on this topic, PUFAs could be an option for controlling inflammatory to patients undergoing dialysis. However, the evidence is not strong enough, especially with regards to the results of hs-CRP and IL-6. For practical recommendation, we anticipate further studies investigating in this topic to further elucidate how PUFAs reduce inflammatory among patients receiving dialysis.

## Figures and Tables

**Table 1:** Characteristics of the included randomized clinical trials.

Author	Location	Inclusion	Treatments	Patients	Mean Age	Sex (M/F)	Dialysis Period
		Year					
Asemi	Iran	2014	1. ω-3	30	55.2	20/10	3.6
[10]			2. αT	30	61.2	20/10	3.5
			3. ω-3 + αT	30	54.9	20/10	3.4
			4. Placebo	30	59.9	20/10	3.4
Bowden	USA	NR	1. ω-3	18	57.2	11/7	1.5
[11]			2. corn oil	15	64.3	8/7	2.8
Daud	USA	NR	1. ω-3	28	59	20/11	3.6
[12]			2. placebo	27	58	12/20	3.3
Ewers	Denmark	2007	1. ω-3	14	64.6	30/10	NR
[13]			2. No supplement	14	64.6	30/10	NR
Gharekhani	Iran	NR	1. ω-3	25	56.8	12/13	5
[14,15,16,17]			2. paraffin (placebo)	20	57.2	8/12	6
Harving	Denmark	NR	1. ω-3	83	65.5	55/28	4
[18]			2. Olive oil	79	68	51/28	3.6
Himmelifarb	USA	2008 to	1. ω-3	31	58	23/8	2.1
[19]		2011	2. placebo	32	61.2	17/15	2.6
Hung	USA	2008 to	1. ω-3	17	50	14/3	4.2
[20]		2011	2. placebo	17	53	13/4	3.6
Khalatbari	Iran	NR	1. ground flaxseed	15	54	10/5	2.6
Soltani [21]			2. Usual diet	15	54.5	6/9	2.8
Kooshki	Iran	NR	1. ω-3	17	50	10/7	1.75
[22]			2. placebo	17	50	11/6	2.3
Lemos	Brazil	NR	1. flaxseed oil + αT	70	55.7	39/31	2.4
[24]			2. mineral oil + αT	75	58.3	46/29	2.9
Lee	Korea	2012	1. ω-3	8	60	2/6	NR
[23]			2. Olive oil	7	64	3/4	NR
Mirfatahi	Iran	NR	1. flaxseed oil	17	68	12/5	4.4
[25]			2. medium-chain	17	59	10/7	4.6
			triglycerides oil				
Naini	Iran	NR	1. ω-3	20	57.7	11/9	NR
[26]			2. placebo	20	59.3	12/8	NR
Poulia	Greece	NR	1. ω-3 + αT	22	51	16/9	9.4
[27]			2. αT	23	51	16/9	
Rodhe	Sweden	NR	1. sea buckthorn + vit-E	24	62	29/16	NR
[28]			2. Coconut oil	21	62	29/16	NR
Saifullah	USA	NR	1. ω-3	15	58	11/4	NR
[29]			2. placebo	8	57	7/1	NR
Zakaria	Egypt	NR	1. ω-3 + vit-E	20	50.2	12/8	4
[30]			2. Placebo	20	46.4	11/9	4.5

CRP, C-reactive protein; hs-CRP, high-sensitivity C-reactive protein; IL-6, interleukin-6; NR, no report; vit-E, vitamin E; αT, alpha-tocopherol; ω-3, omega-3 fatty acids.

**Table 2:** Quality of the included randomized clinical trials.

Study	Randomization	Concealment	Blinding	Follow-Up	Loss	Type of	Relevant	Quality
				Duration	Follow-Up	Analysis	Outcomes	Judgement
Asemi	Computer generated	Yes	Double-blind	12 weeks	0	ITT	hs-CRP	Low risk
Bowden	4-block permuted randomization	No	Double-blind	26 weeks	7	PP	hs-CRP	High risk
Daud	NR	NR	Triple-blind	26 weeks	2	ITT	CRP	Moderate
Ewers	Computer generated	NR	Single-blind	6 weeks	10	PP	CRP	High risk
Gharekhani	Blocked randomization	NR	Single-blind	16 weeks	9	PP	CRP, IL-6	High risk
Harving	NR	NR	NR	12 weeks	44	PP	hs-CRP	High risk
Himmelifarb	4-block permuted randomization	NR	Double-blind	8 weeks	0	ITT	CRP, IL-6	Moderate
Hung	Randomized in 1:1 ratio	NR	Double-blind	12 weeks	4	PP	hs-CRP, IL-6	Moderate
KhalatbariSoltani	NR	NR	Unblind	8 weeks	8	PP	CRP	High risk
Kooshki	Blocked randomization	Yes	Double-blind	10 weeks	0	ITT	CRP, IL-6	Low risk
Lee	Random number table	NR	Double-blind	12 weeks	0	PP	CRP	Moderate
Lemos	NR	Yes	Double-blind	7 weeks	22	ITT	CRP	High risk
Mirfatahi	Blocked randomization	NR	Double-blind	8 weeks	0	PP	hs-CRP	Moderate
Naini	NR	Yes	Double-blind	8 weeks	0	ITT	CRP, IL-6	Moderate
Poulia	Flip coin	NR	Single-blind	4 weeks	8	PP	CRP	High risk
Rodhe	NR	Yes	Double-blind	8 weeks	21	PP	hs-CRP	High risk
Saifullah	Computer generated	Yes	Double-blind	12 weeks	3	PP	CRP	Moderate
Zakaria	Flip coin	NR	Double-blind	16 weeks	0	PP	hs-CRP	Moderate

CRP, C-reactive protein; hs-CRP, high-sensitivity C-reactive protein; IL-6, interleukin-6; ITT, intention to treat; NR, no report; PP, per protocol.

## Supplementary Materials

Supplementary materials can be found at https://www.mdpi.com/1422-0067/20/15/3645/s1.

## Abbreviations

CI	confidence interval
CRP	C-reactive protein
hs-CRP	high-sensitivity C-reactive protein
IQR	interquartile range
IL-6	interleukin-6
MCT	medium chain triglyceride
NF-κB	nuclear factor kappa B
PUFA	polyunsaturated fatty acids
RCT	randomized clinical trial
SD	standard deviation.
SE	standard error
SUCRA	surface under the cumulative ranking curve
WMD	weighted mean difference
