# Supplementary material for: Efficacy of Polyunsaturated Fatty Acids on Inflammatory Markers in Patients Undergoing Dialysis: A Systematic Review with Network Meta-Analysis of Randomized Clinical Trials
Source: Int J Mol Sci. 2019 Jul 25;20(15):3645. doi: 10.3390/ijms20153645 (PMC6695890; doi:10.3390/ijms20153645)
Supplement: Supplementary file 1 [file ijms-20-03645-s001.pdf]

Efficacy of Polyunsaturated Fatty Acids on Inflammatory Markers in Patients Undergoing Dialysis:

a Systematic Review with Network Meta-Analysis of Randomized Clinical Trials

(Supplementary Files)

Supplementary File

Supplementary File 1. Head-to-head comparisons of CRP

Supplementary File 2. Ranking probability and SUCRA of CRP

Supplementary File 3. Small study bias in network meta-analysis of CRP

Supplementary File 4. Head-to-head comparisons of hs-CRP

Supplementary File 5. Ranking probability and SUCRA of hs-CRP

Supplementary File 6. Inconsistency test for network meta-analysis of hs-CRP

Supplementary File 7. Small study bias in network meta-analysis of hs-CRP

Supplementary File 8. Head-to-head comparisons of IL-6

Supplementary File 9. Ranking probability and SUCRA of IL-6

Supplementary File 10. Small study bias in network meta-analysis of IL-6

Supplementary File 11. Head-to-head comparisons of albumin

Supplementary File 12. Ranking probability and SUCRA of albumin

Supplementary File 13. Inconsistency test for network meta-analysis of hs-CRP

Supplementary File 14. Small study bias in network meta-analysis of albumin

Supplementary File 15. Detection of Regions on the outcomes of CRP

Supplementary File 16. Detection of Regions on the outcomes of hs-CRP

Supplementary File 17. Cluster plot of SUCRA for CRP and hs-CRP

Supplementary File 18. Search strategy

Supplementary File 1 to 3

Outcome of C-reactive protein

Supplementary File 1

Head-to-head comparisons of CRP

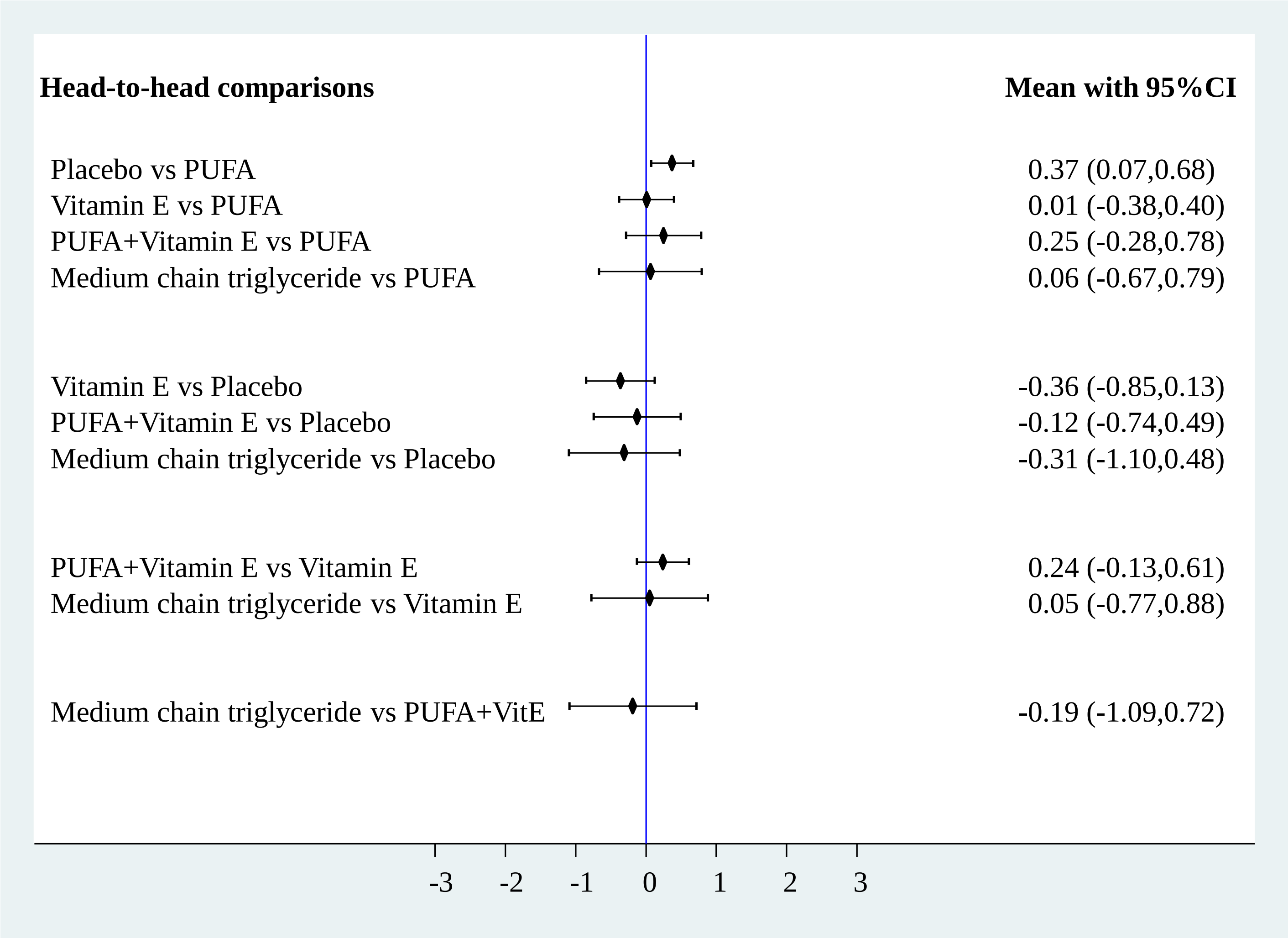

Supplementary File 2

Ranking probability and SUCRA of CRP

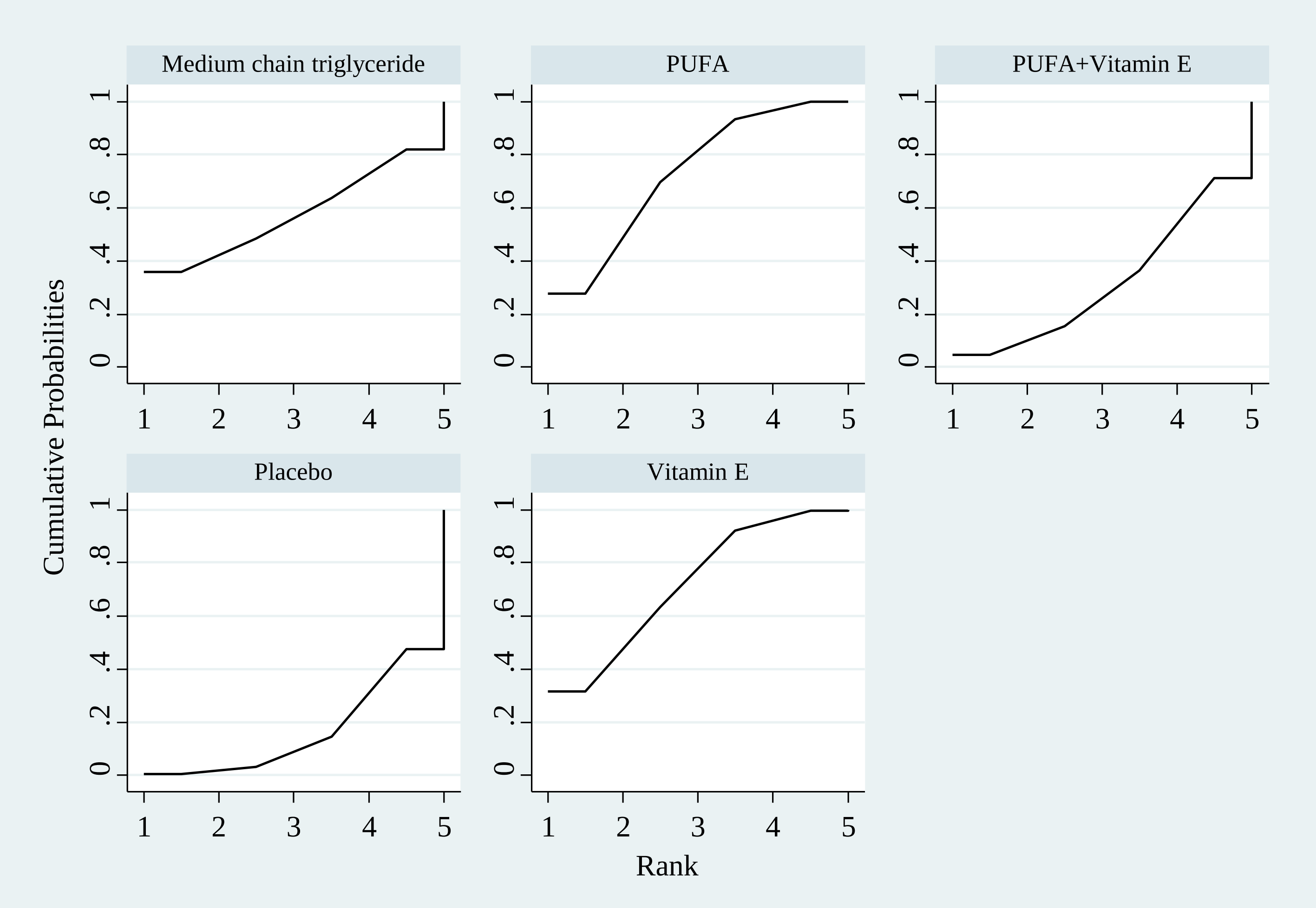

. sucra prob*, labels("PUFA" "Placebo" "Vitamin E" "PUFA+Vitamin E" "Medium chain triglyceride")

Treatment	SUCRA	PrBest	MeanRank
PUFA	72.7	27.7	2.1
Placebo	16.3	0.3	4.3
Vitamin E	71.6	31.6	2.1
PUFA+Vitamin E	31.9	4.7	3.7
Medium chain triglyceride	57.4	35.7	2.7

Supplementary File 3

Small study bias in network meta-analysis of CRP

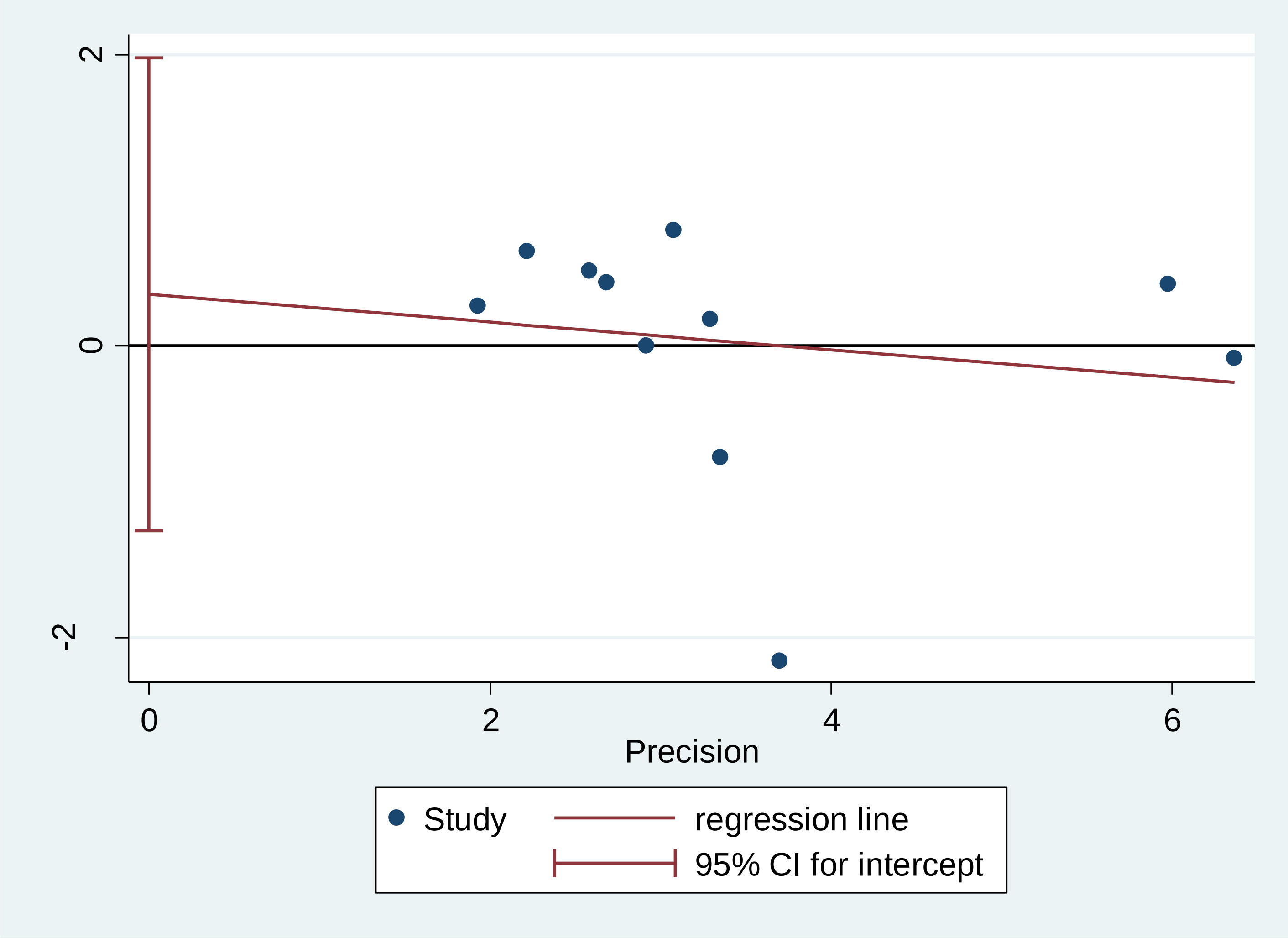


```
. metabias _ES_CEN seES , egger graph

Number of studies =  11                    Root MSE      =    .875
------------------------------------------------------------------------------
    Std_Eff |      Coef.   Std. Err.      t    P>|t|     [95% Conf. Interval]
-------------+----------------------------------------------------------------
      slope |  -.0953048   .1930426    -0.49   0.633    -.5319974    .3413878
       bias |   .3555706   .7185349     0.49   0.633    -1.269868    1.981009
------------------------------------------------------------------------------

Test of H0: no small-study effects          P = 0.633
```

Supplementary File 4 to 7

Outcomes of high sensitive C-reactive protein

Supplementary File 4

Head-to-head comparisons of hs-CRP

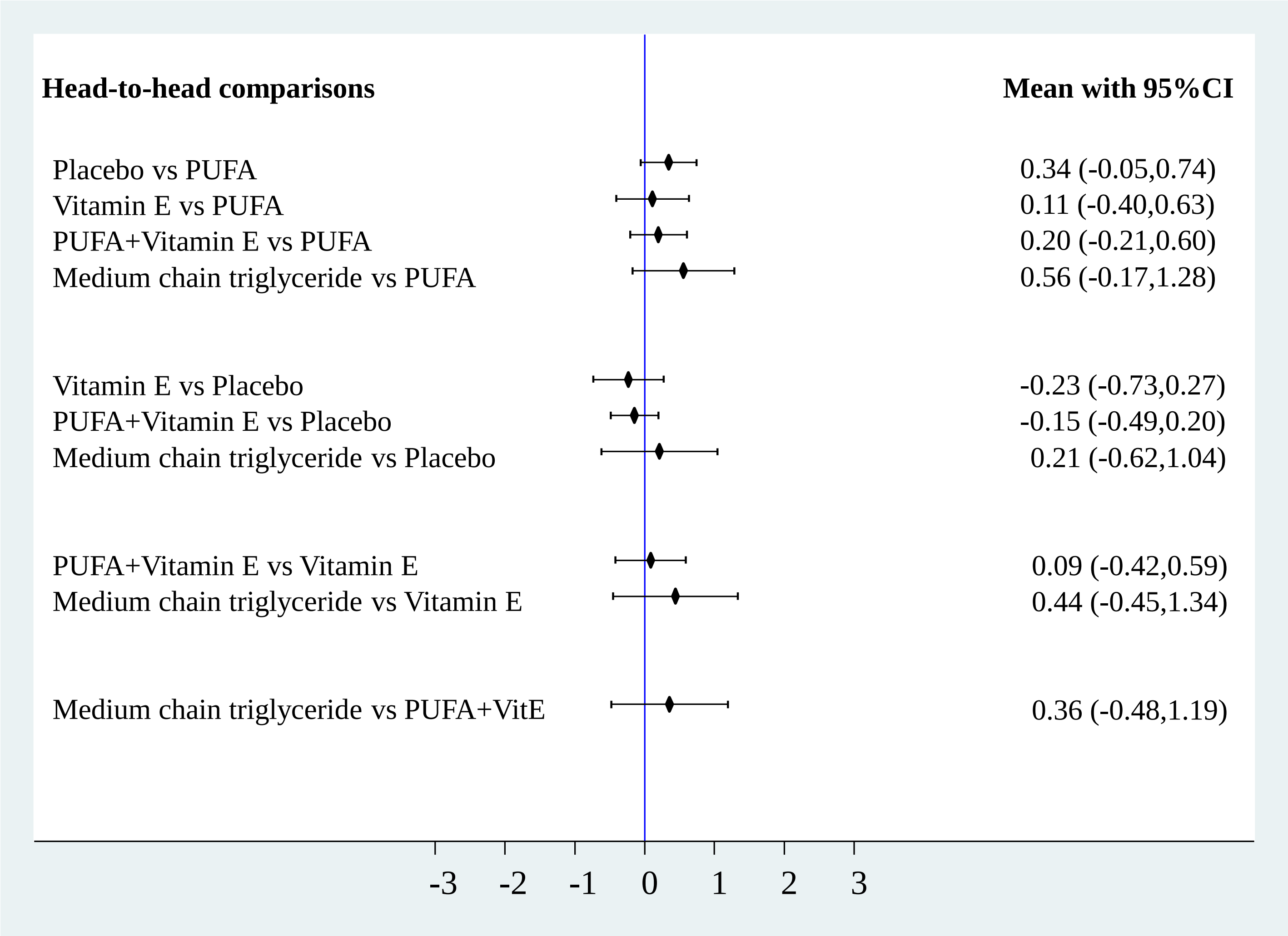

Supplementary File 5

Ranking probability and SUCRA of hs-CRP

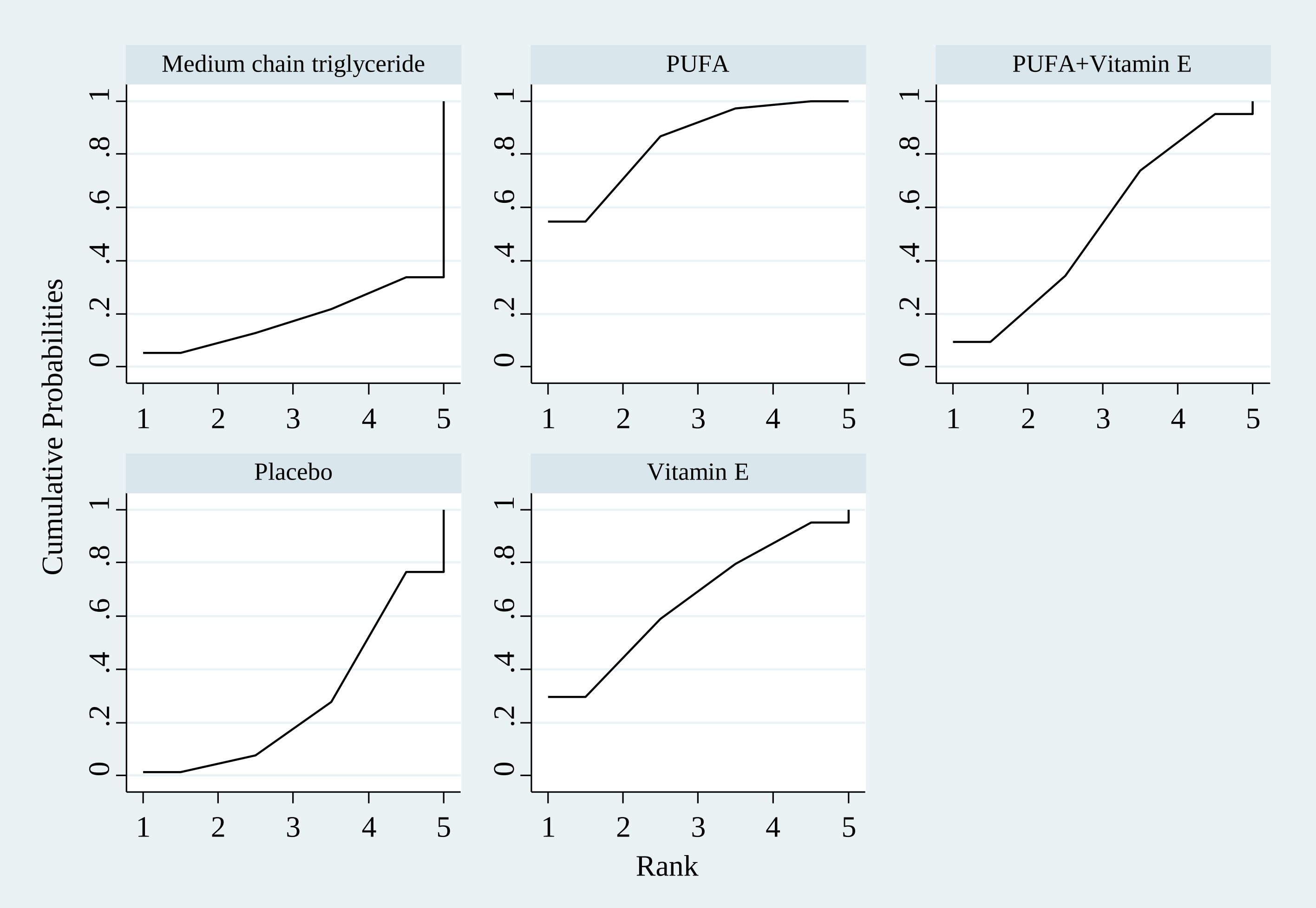

. sucra prob*, labels("PUFA" "Placebo" "Vitamin E" "PUFA+Vitamin E" "Medium chain triglyceride")

Treatment	SUCRA	PrBest	MeanRank
PUFA	84.6	54.7	1.6
Placebo	28.2	1.2	3.9
Vitamin E	65.7	29.4	2.4
PUFA+Vitamin E	53.1	9.4	2.9
Medium chain triglyceride	18.4	5.3	4.3

Supplementary File 6

Inconsistency test for network meta-analysis of hs-CRP

```
. network meta i, luades

initial:       log likelihood = -12.736872
rescale:       log likelihood = -11.490398
rescale eq:    log likelihood = -10.661025
Iteration 0:   log likelihood = -10.661025
Iteration 1:   log likelihood = -10.368251  (not concave)
Iteration 2:   log likelihood = -10.021288
Iteration 3:   log likelihood = -9.9930264
Iteration 4:   log likelihood = -9.9924889
Iteration 5:   log likelihood = -9.9924888

Multivariate meta-analysis
Variance-covariance matrix = proportional .5*I(4)+.5*J(4,4,1)
Method = reml                                   Number of dimensions     =      4
Restricted log likelihood = -9.9924888          Number of observations   =      6
------------------------------------------------------------------------------
             |      Coef.   Std. Err.      z    P>|z|     [95% Conf. Interval]
-------------+----------------------------------------------------------------
_y_B         |
       _cons |   .4690762   .2054935     2.28   0.022     .0663163     .871836
-------------+----------------------------------------------------------------
_y_C         |
       _cons |   .1090441   .2387646     0.46   0.648     -.358926    .5770142
-------------+----------------------------------------------------------------
_y_D         |
      groupB |   .4761785   .3177915     1.50   0.134    -.1466813    1.099038
       _cons |   .0624081   .2030203     0.31   0.759    -.3355044    .4603206
-------------+----------------------------------------------------------------
_y_E         |
       _cons |   .5561661   .3503694     1.59   0.112    -.1305454    1.242877
------------------------------------------------------------------------------

Estimated between-studies SDs and correlation matrix:
            SD        _y_B        _y_C        _y_D        _y_E
_y_B  1.681e-08          1           .           .           .
_y_C  1.681e-08         .5           1           .           .
_y_D  1.681e-08         .5          .5           1           .
_y_E  1.681e-08         .5          .5          .5           1

Testing for inconsistency:
 ( 1)  [_y_D]groupB = 0

           chi2(  1) =    2.25
         Prob > chi2 =    0.1340
```

Supplementary File 7

Small study bias in network meta-analysis of hs-CRP

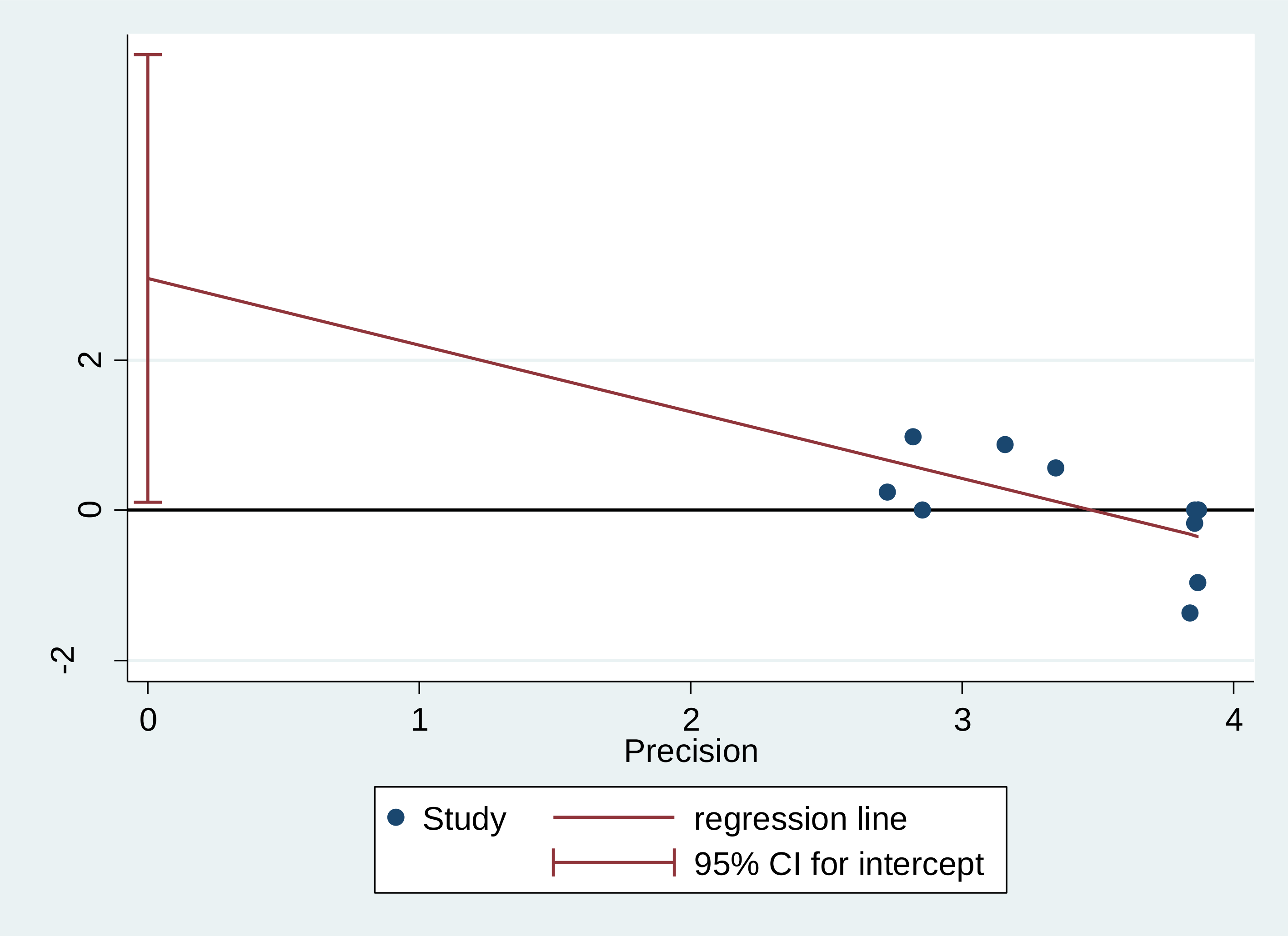


```
. metabias _ES_CEN seES , egger graph

Number of studies =  11                    Root MSE      =     .582
------------------------------------------------------------------------------
     Std_Eff |      Coef.     Std. Err.       t     P>|t|     [95% Conf. Interval]
-------------+----------------------------------------------------------------
       slope |  -.8856047   .3767878  -2.35   0.043  -1.737958   -.0332515
        bias |  3.080732   1.315908     2.34   0.044    .1039413  6.057522
------------------------------------------------------------------------------

Test of H0: no small-study effects          P = 0.044
```

Supplementary File 8 to 10
Outcomes of Interleukin-6

Supplementary File 8

Head-to-head comparisons of IL-6

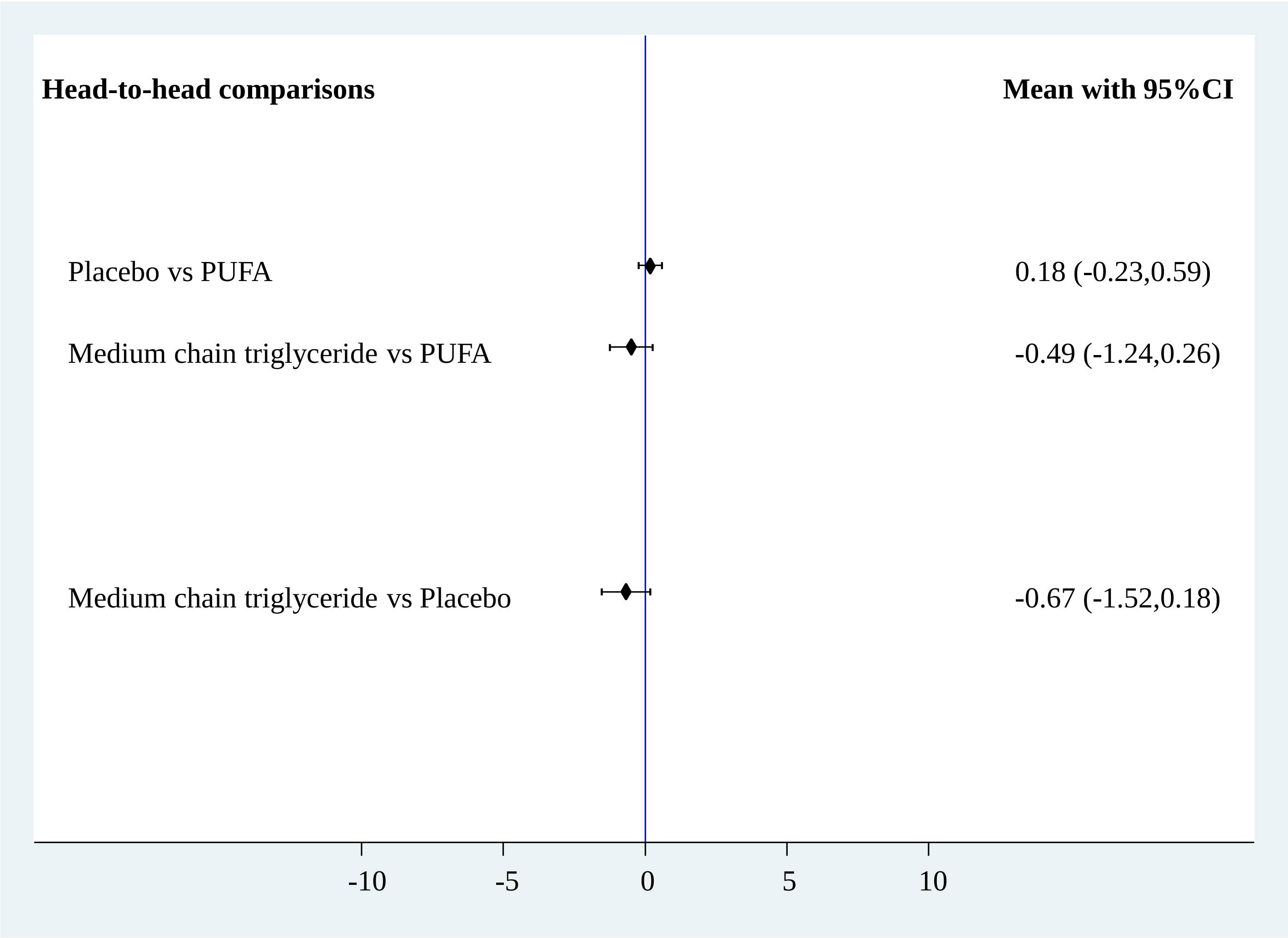

Supplementary File 9
Ranking probability and SUCRA of IL-6

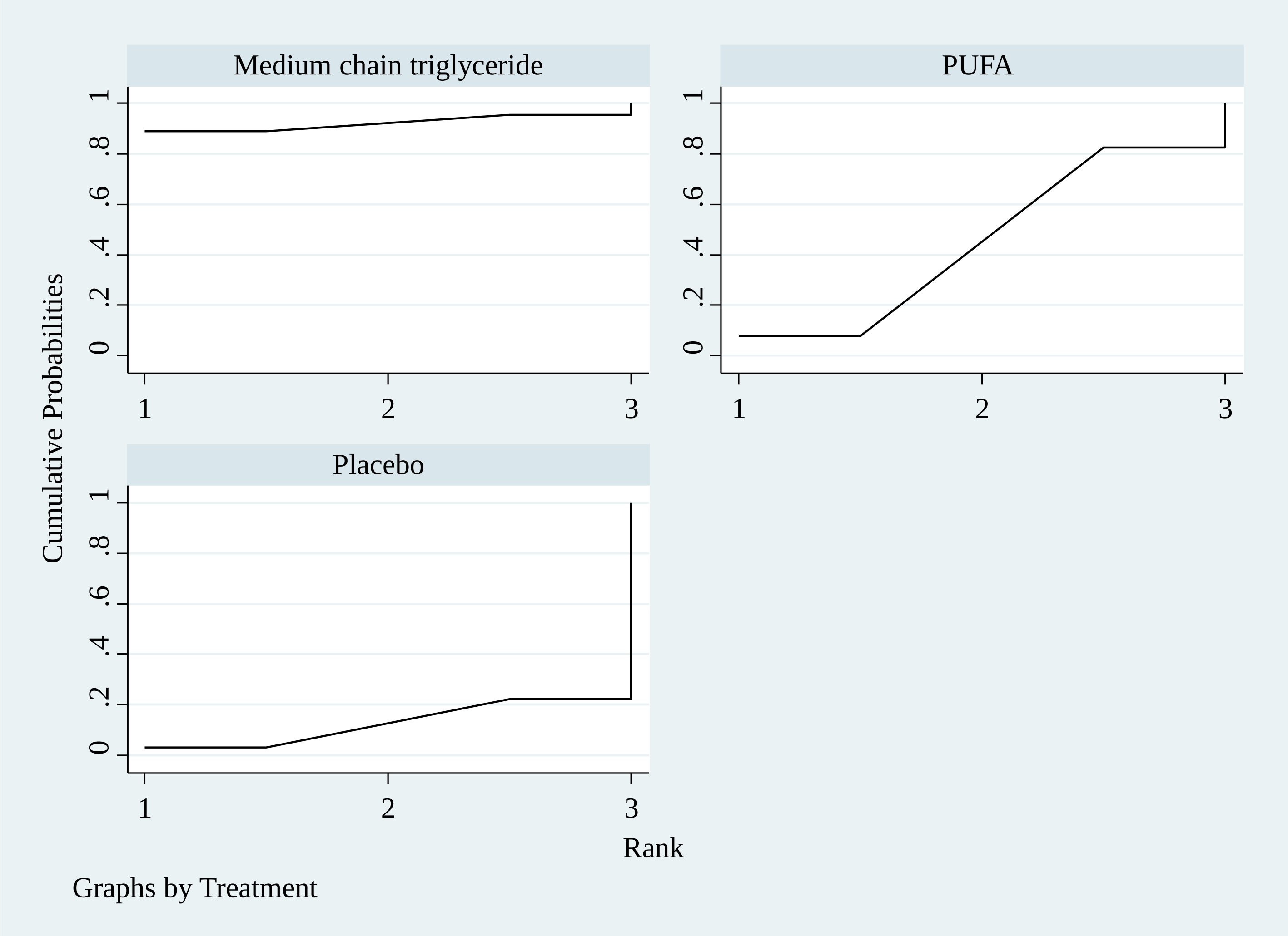

. sucra prob*, labels("PUFA" "Placebo" "Medium chain triglyceride")

Treatment	SUCRA	PrBest	MeanRank
PUFA	45.2	8.0	2.1
Placebo	12.7	3.2	2.7
Medium chain triglyceride	92.0	88.8	1.2

Supplementary File 10

Small study bias in network meta-analysis of IL-6

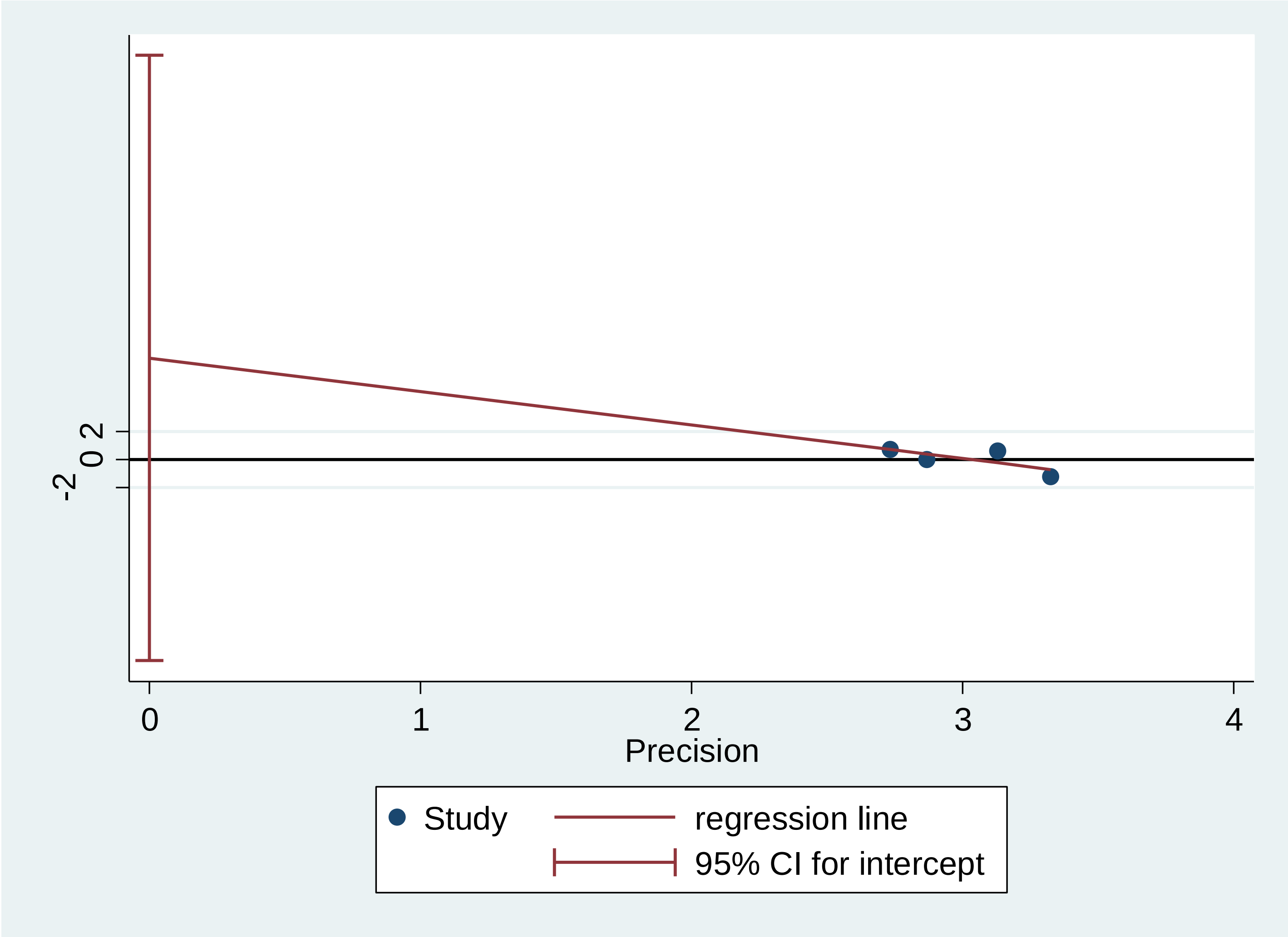


```
. metabias _ES_CEN seES , egger graph

Number of studies =  4                     Root MSE      =   .7567
------------------------------------------------------------------------------
   Std_Eff |      Coef.   Std. Err.      t    P>|t|     [95% Conf. Interval]
-------------+----------------------------------------------------------------
     slope |  -2.373224   1.648212    -1.44   0.287    -9.464908    4.718461
      bias |   7.184133   4.983411     1.44   0.286    -14.25776    28.62602
------------------------------------------------------------------------------

Test of H0: no small-study effects          P = 0.286
```

Supplementary File 11 to 14

Outcomes of Albumin

Supplementary File 11

Head-to-head comparisons of albumin

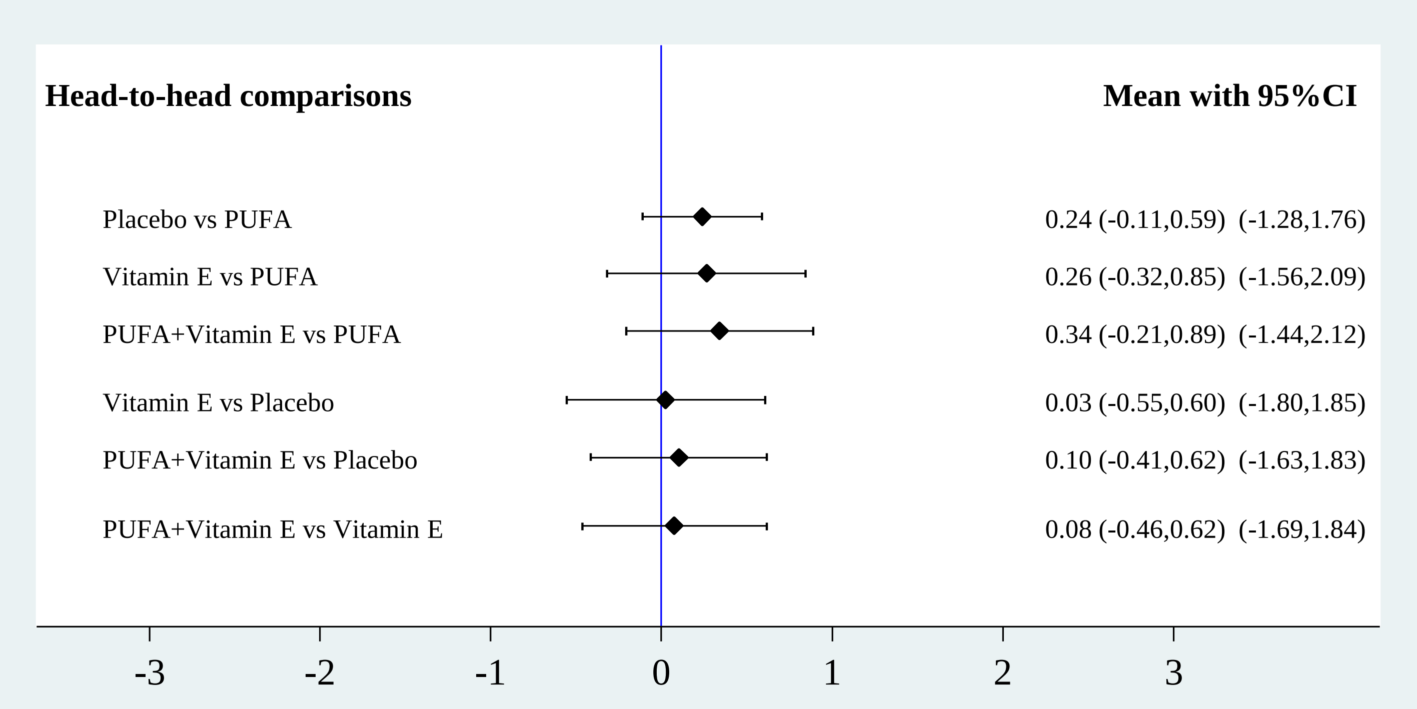

Supplementary File 12

Ranking probability and SUCRA of albumin

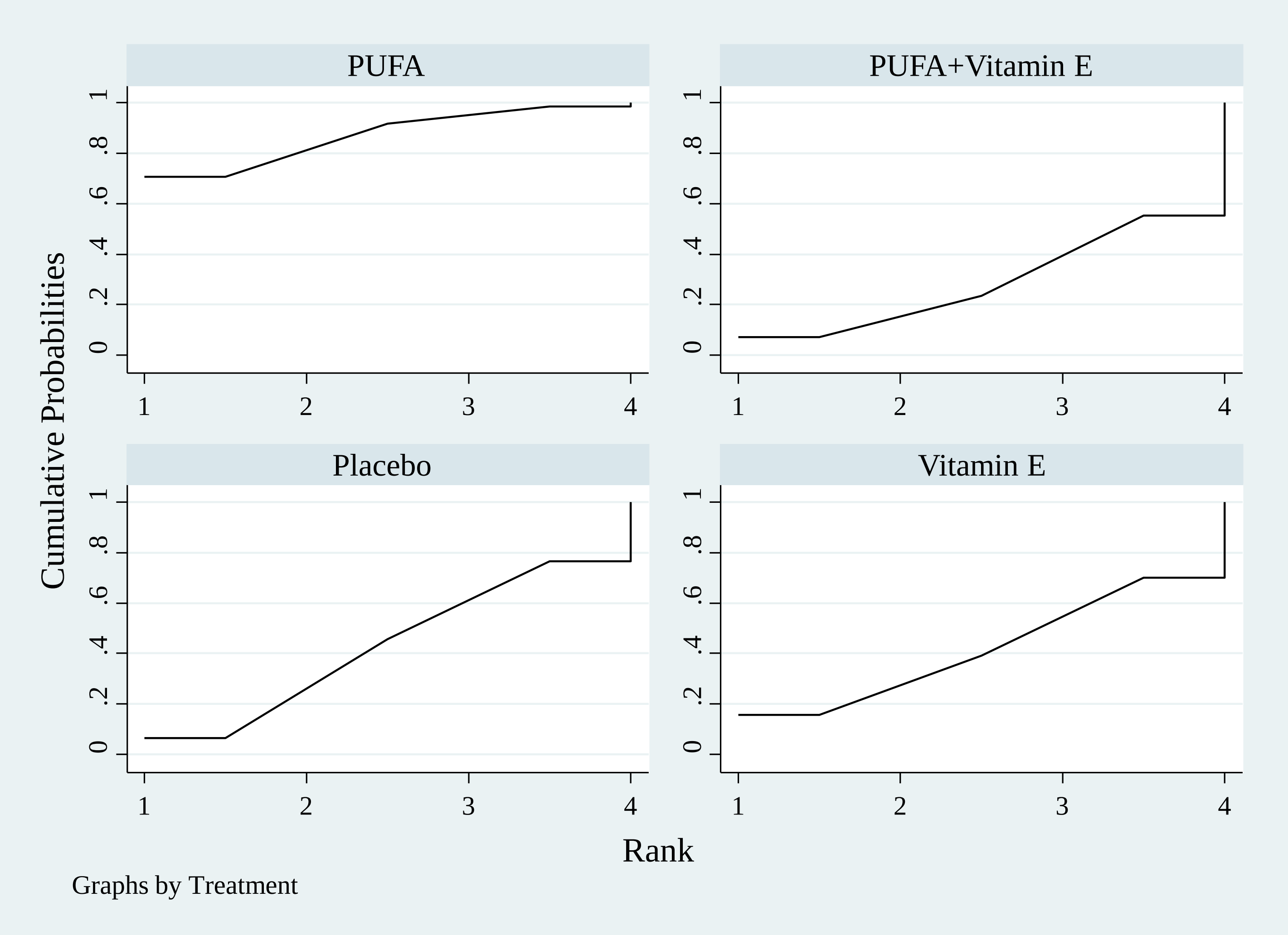


```
. sucra prob*, labels("PUFA" "Placebo" "Vitamin E" "PUFA+Vitamin E")
```

Treatment	SUCRA	PrBest	MeanRank
PUFA	86.9	70.6	1.4
Placebo	42.8	6.4	2.7
Vitamin E	41.7	15.7	2.7
PUFA+Vitamin E	28.6	7.2	3.1

Supplementary File 13

Inconsistency test for network meta-analysis of albumin

```
. network meta i, luades

initial:       log likelihood = -16.443995
rescale:       log likelihood = -14.678721
rescale eq:    log likelihood = -13.884903
Iteration 0:   log likelihood = -13.884903
Iteration 1:   log likelihood = -13.555383
Iteration 2:   log likelihood = -13.546198
Iteration 3:   log likelihood = -13.546175
Iteration 4:   log likelihood = -13.546175

Multivariate meta-analysis
Variance-covariance matrix = proportional .5*I(3)+.5*J(3,3,1)
Method = reml                                   Number of dimensions    =     3
Restricted log likelihood = -13.546175          Number of observations  =     9
------------------------------------------------------------------------------
             |      Coef.   Std. Err.      z    P>|z|     [95% Conf. Interval]
-------------+----------------------------------------------------------------
_y_B         |
       _cons |   .2308547   .1478359     1.56   0.118    -.0588983    .5206077
-------------+----------------------------------------------------------------
_y_C         |
       _cons |   .4192418   .2803931     1.50   0.135    -.1303187    .9688022
-------------+----------------------------------------------------------------
_y_D         |
      groupB |   .2329741    .439844     0.53   0.596    -.6291043    1.095052
      groupC |   1.083716   .4673996     2.32   0.020     .1676299    1.999803
       _cons |    .053441   .2740923     0.19   0.845    -.4837701    .5906521
------------------------------------------------------------------------------

Estimated between-studies SDs and correlation matrix:
             SD        _y_B        _y_C        _y_D
_y_B  .16339678           1           .           .
_y_C  .16339678          .5           1           .
_y_D  .16339678          .5          .5           1

Testing for inconsistency:
 ( 1)  [_y_D]groupB = 0
 ( 2)  [_y_D]groupC = 0

           chi2(  2) =    5.38
         Prob > chi2 =    0.0680
```

Supplementary File 14

Small study bias in network meta-analysis of albumin

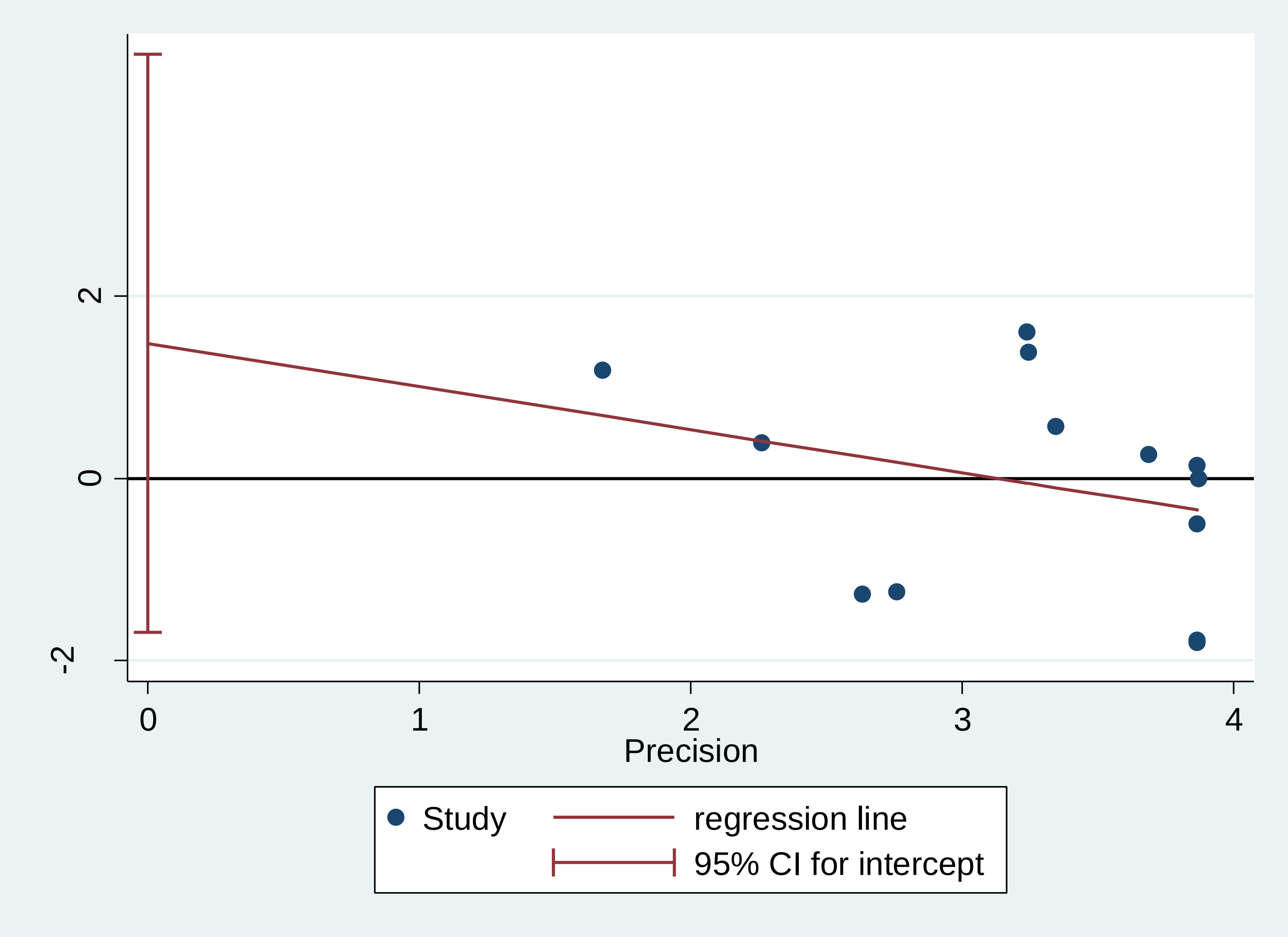

. metabias _ES_CEN seES , egger graph

Number of studies = 14 Root MSE = 1.109

Std_Eff	Coef.	Std. Err.	t	P>|t|	[95% Conf. Interval]	
slope	-.4728177	.4332609	-1.09	0.297	-1.416812	.4711767
bias	1.481466	1.455748	1.02	0.329	-1.690337	4.653269

Test of H0: no small-study effects P = 0.329

Supplementary File 15 to 16

Detection of Regions on the outcomes of C-reactive protein and high sensitive C-reactive protein

Supplementary File 15

Detection of Regions on the outcomes of C-reactive protein

```
. network meta c, regress(Asia America Europe) eform(Mean difference)
Command is: mvmeta _y _S Asia America Europe  , eform(Mean difference)
initial:       log likelihood = -8.3767857
rescale:       log likelihood =  -7.529987
rescale eq:    log likelihood = -6.5780492
Iteration 0:   log likelihood = -6.5780492  (not concave)
Iteration 1:   log likelihood = -6.3847531  (not concave)
Iteration 2:   log likelihood =  -6.382383
Iteration 3:   log likelihood = -6.3445826
Iteration 4:   log likelihood = -6.3435081
Iteration 5:   log likelihood = -6.3434965
Iteration 6:   log likelihood = -6.3434965

Multivariate meta-analysis
Variance-covariance matrix = proportional .5*I(4)+.5*J(4,4,1)
Method = reml                              Number of dimensions    =     4
Restricted log likelihood = -6.3434965        Number of observations  =    11
------------------------------------------------------------------------------
             |Mean difference   Std. Err.      z    P>|z|     [95% Conf. Interval]
-------------+----------------------------------------------------------------
_y_B         |
        Asia |    .954335    .411708    -0.11   0.914     .4097184    2.222881
     America |   .5749935   .2594293    -1.23   0.220      .237472    1.392238
       _cons |   1.783514   .6901929     1.50   0.135     .8353616    3.807839
-------------+----------------------------------------------------------------
_y_C         |
        Asia |   1.357536   42.95153     0.01   0.992     1.59e-27    1.16e+27
      Europe |     1.1617   36.75095     0.00   0.996     1.37e-27    9.85e+26
       _cons |   .8519952   26.95295    -0.01   0.996     1.01e-27    7.22e+26
-------------+----------------------------------------------------------------
_y_D         |
     America |   1.161048   36.73196     0.00   0.996     1.37e-27    9.87e+26
       _cons |   1.022543   .3447171     0.07   0.947     .5281131    1.979869
-------------+----------------------------------------------------------------
_y_E         |
       _cons |    1.06683   .3660272     0.19   0.850      .544565    2.089973
------------------------------------------------------------------------------

Estimated between-studies SDs and correlation matrix:
           SD        _y_B        _y_C        _y_D        _y_E
_y_B  5.097e-09          1           .           .           .
_y_C  5.097e-09         .5           1           .           .
_y_D  5.097e-09         .5          .5           1           .
_y_E  5.097e-09         .5          .5          .5           1
```

Supplementary File 16

Detection of Regions on the outcomes of high sensitive C-reactive protein

```
. network meta c, regress(Asia America Europe) eform(Mean difference)
Command is: mvmeta _y _S Asia America Europe  , eform(Mean difference)
initial:       log likelihood = -5.7716745
rescale:       log likelihood = -5.6813907
rescale eq:    log likelihood = -5.3667652
Iteration 0:   log likelihood = -5.3667652
Iteration 1:   log likelihood =  -5.333843  (not concave)
Iteration 2:   log likelihood = -5.3337078
Iteration 3:   log likelihood = -5.3335849
Iteration 4:   log likelihood = -5.3335849

Multivariate meta-analysis
Variance-covariance matrix = proportional .5*I(4)+.5*J(4,4,1)
Method = reml                                   Number of dimensions    =     4
Restricted log likelihood = -5.3335849          Number of observations  =     6
------------------------------------------------------------------------------
             |Mean difference   Std. Err.      z    P>|z|     [95% Conf. Interval]
-------------+----------------------------------------------------------------
_y_B         |
        Asia |   1.247846   39.45615     0.01   0.994     1.52e-27    1.02e+27
     America |   1.646605   52.06696     0.02   0.987     2.00e-27    1.36e+27
       _cons |    .990318   31.31072    -0.00   1.000     1.21e-27    8.09e+26
-------------+----------------------------------------------------------------
_y_C         |
       _cons |   1.001552   .4278986     0.00   0.997     .4335207     2.31386
-------------+----------------------------------------------------------------
_y_D         |
        Asia |   .9885095   31.25598    -0.00   1.000     1.20e-27    8.12e+26
     America |   1.553719   49.12946     0.01   0.989     1.89e-27    1.28e+27
       _cons |   1.008552   31.88712     0.00   1.000     1.23e-27    8.24e+26
-------------+----------------------------------------------------------------
_y_E         |
       _cons |   1.743973   .8519281     1.14   0.255     .6694672     4.54308
------------------------------------------------------------------------------

Estimated between-studies SDs and correlation matrix:
            SD        _y_B        _y_C        _y_D        _y_E
_y_B  .34039961          1           .           .           .
_y_C  .34039961         .5           1           .           .
_y_D  .34039961         .5          .5           1           .
_y_E  .34039961         .5          .5          .5           1
```

Supplementary File 17

Cluster plot of SUCRA for CRP and hs-CRP

Supplementary File 17

Cluster plot of SUCRA for CRP and hs-CRP

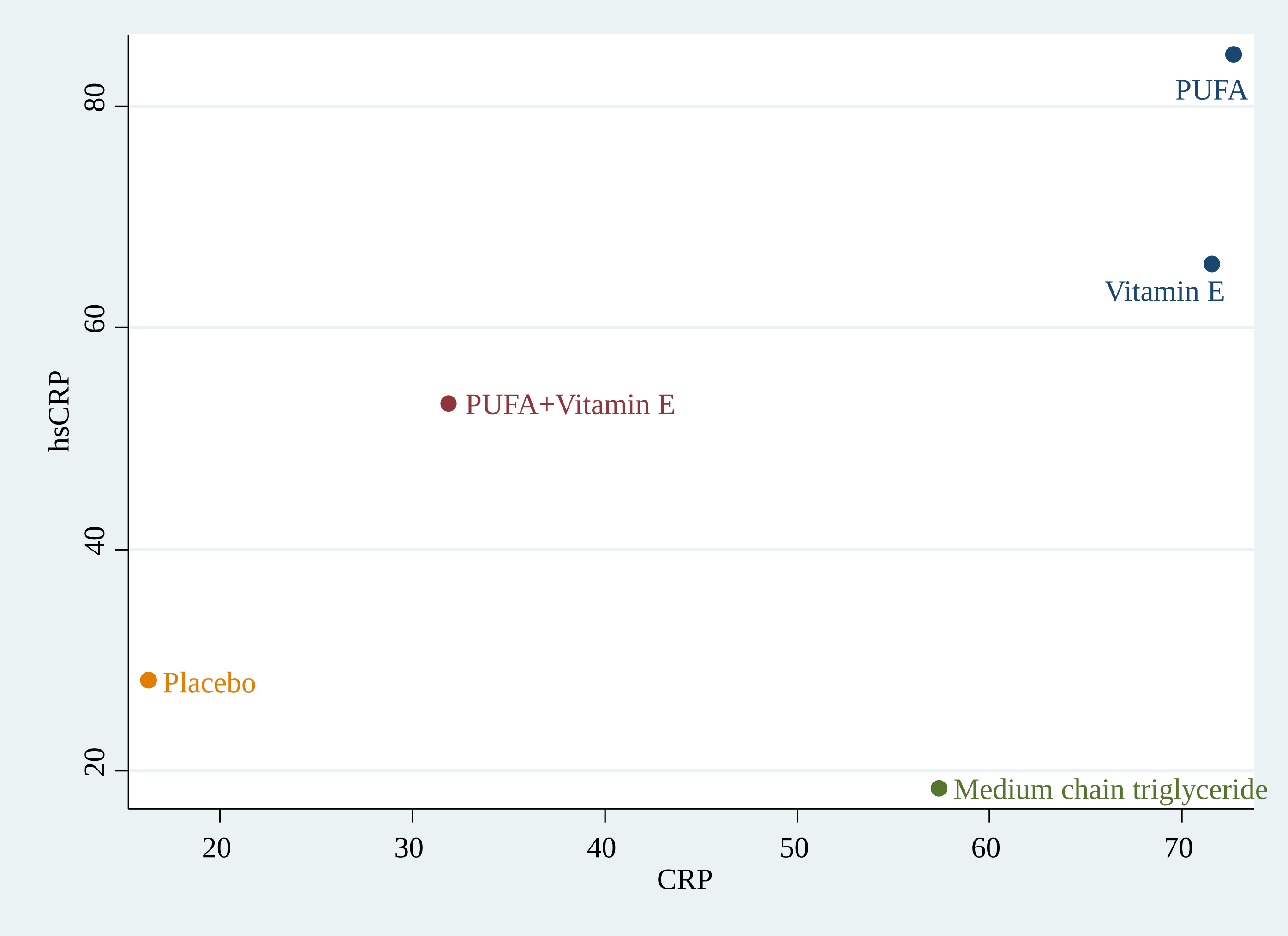

Supplementary File 18
Search strategy

Supplementary File 18
Search strategy

Embase:
('dialysis'/exp OR 'acute dialysis' OR 'coil dialysis' OR 'dialysis' OR 'flow dialysis' OR 'hemodialysis'/exp OR 'blood dialysis' OR 'chronic haemodialysis' OR 'chronic hemodialysis' OR 'chronic intermittent haemodialysis' OR 'chronic intermittent hemodialysis' OR 'dialysis center' OR 'dialysis, blood' OR 'extracorporeal blood cleansing' OR 'extracorporeal dialysis' OR 'haemodialysis' OR 'haemodialysis center' OR 'haemodialysis centre' OR 'haemodialysis department' OR 'haemodialysis unit' OR 'haemodialysis units, hospital' OR 'hemodialyse' OR 'hemodialysis' OR 'hemodialysis center' OR 'hemodialysis department' OR 'hemodialysis unit' OR 'hemodialysis units, hospital' OR 'hemorenodialysis' OR 'hemotrialysate' OR 'intermittent chronic haemodialysis' OR 'intermittent chronic hemodialysis' OR 'intermittent haemodialysis' OR 'intermittent hemodialysis' OR 'renal dialysis') AND ('omega 3' OR omega3 OR 'omega-3 fatty acids' OR 'omega-3 fatty acid' OR 'omega3 fatty acids' OR 'omega3 fatty acid' OR docosahexaenoic OR dha OR eicosapentaenoic OR epa OR polyunsaturated OR 'polyunsaturated fatty acids' OR 'polyunsaturated fatty acid'/exp OR 'fatty acid polyunsaturation' OR 'fatty acid, polyunsaturated' OR 'poly unsaturated fatty acid' OR 'polyunsaturated fat' OR 'polyunsaturated fatty acid' OR lcpufa* OR pufa* OR 'ω 3' OR ω3 OR 'n3 fatty acid' OR 'n3 fatty acids' OR 'n-3 fatty acid' OR 'n-3 fatty acids' OR 'omega-6 fatty acids' OR 'omega-6 fatty acid' OR 'omega6 fatty acids' OR 'omega6 fatty acid')

PubMed:
(dialysis[ti] OR hemodialysis[ti]) AND (omega 3 OR omega3 OR omega-3 fatty acids OR omega-3 fatty acid OR omega3 fatty acids OR omega3 fatty acid OR docosahexaenoic OR dha OR eicosapentaenoic OR epa OR polyunsaturated OR polyunsaturated fatty acids OR polyunsaturated fatty acid OR LCPUFA* OR PUFA* OR ω-3 OR ω3 OR n3 fatty acid OR n3 fatty acids OR n-3 fatty acid OR n-3 fatty acids OR omega-6 fatty acids OR omega-6 fatty acid OR omega6 fatty acids OR omega6 fatty acid)

Web of Science:
TOPIC: (dialysis OR hemodialysis) AND TOPIC: (omega 3 OR omega3 OR omega-3 fatty acids OR omega-3 fatty acid OR omega3 fatty acids OR omega3 fatty acid OR docosahexaenoic OR dha OR eicosapentaenoic OR epa OR polyunsaturated OR polyunsaturated fatty acids OR polyunsaturated fatty acid OR LCPUFA* OR PUFA* OR ω-3 OR ω3 OR n3 fatty acid OR n3 fatty acids OR n-3 fatty acid OR n-3 fatty acids OR omega-6 fatty acids OR omega-6 fatty acid OR omega6 fatty acids OR omega6 fatty acid)
